# Racial and ethnic disparities in type 2 diabetes: does visceral fat play a role? Evidence from the MESA study

**DOI:** 10.1038/s41366-026-02079-2

**Published:** 2026-04-08

**Authors:** Tong Xia, Roch A. Nianogo, QingZhao Yu, Tamara Horwich, Preethi Srikanthan, Kosuke Inoue, Matthew Allison, Zuo-Feng Zhang, Moyses Szklo, Karol E. Watson, Liwei Chen

**Affiliations:** 1 Department of Epidemiology, Fielding School of Public Health, University of California Los Angeles, Los Angeles, CA USA; 2 Channing Division of Network Medicine, Harvard Medical School and Brigham and Women’s Hospital, Boston, MA USA; 3 Department of Nutrition, Harvard T.H. Chan School of Public Health, Boston, MA USA; 4 California Center for Population Research (CCPR), Los Angeles, CA USA; 5 Department of Biostatistics, School of Public Health, Louisiana State University Health Sciences Center-New Orleans, New Orleans, LA USA; 6 Department of Medicine, University of California Los Angeles, Los Angeles, CA USA; 7 Department of Social Epidemiology, Graduate School of Medicine, Kyoto University, Kyoto, Japan; 8 Department of Family Medicine and Public Health, School of Medicine, University of California San Diego, San Diego, CA USA; 9 Department of Epidemiology, Bloomberg School of Public Health, Johns Hopkins University, Baltimore, MD USA

**Keywords:** Risk factors, Type 2 diabetes

## Abstract

**Objective:**

In the United States, racial and ethnic minorities have higher risk of developing type 2 diabetes (T2D) than Whites. One hypothesis is that some minorities (e.g., Hispanics, Asians) have more visceral fat than Whites in a sex-specific manner. We aimed to test this hypothesis by examining to what degree racial differences in T2D were explained by visceral fat in males and females.

**Methods:**

This prospective cohort included 1457 participants (51.2% females) from the Multi-Ethnic Study of Atherosclerosis (MESA) cohort who had visceral fat measured by computed tomography and followed for incident T2D from 2002–2005 to 2020. We assessed associations of race and T2D risk using Cox proportional hazards regressions and estimated associations explained by visceral fat using natural mediation effects, stratified by sex.

**Results:**

Controlling for confounders, compared with White participants, T2D risk was higher in Hispanic females (HR 1.77, 95% CI 1.17–2.69), Chinese females (1.91, 1.15–3.15), Black females (1.59, 1.02–2.49), and Hispanic males (1.82, 1.20–2.76). Estimates for Black males (1.48, 0.92–2.38) and Chinese males (0.86, 0.48–1.55) were not statistically significant. By sex, Hispanic females [mean difference (SE): 22.72 (5.68)] had higher visceral fat (cm^2^) than White females, while Chinese [−77.56 (8.47)] and Black [−57.57 (8.11)] males had lower visceral fat (cm^2^) than White males. Visceral fat explained 23.1% of T2D risk between Hispanic and White females, but not for other racial and sex subgroups.

**Conclusion:**

Visceral fat explained one-fifth of racial and ethnic differences in T2D comparing Hispanic females to White females and may contribute to Hispanic females’ higher T2D risk.

## Introduction

In 2021, 11.6% Americans are estimated to live with diabetes, with 95% having type 2 diabetes (T2D) [1]. Meanwhile, racial disparities in T2D are persistent in the United States (US) [1–3]. The National Diabetes Statistics Report showed from 2017 through 2018, Hispanics had the highest age-adjusted incidence rate of T2D at 9.7 per 1000 persons, followed by Blacks at 8.2, Asians at 7.4, and Whites at 5.0 per 1000 persons in the US [2]. Thus, it is essential to find determinants of T2D to narrow such disparities.

Visceral fat, surrounding abdominal organs, has been considered a leading risk factor for T2D, independent of overall adiposity [4, 5], and more closely associated with T2D than subcutaneous fat [6]. It may be due to visceral fat’s high lipolysis rate [7] and adipocytokine release [8], which contributes to insulin resistance, resulting in T2D.

Studies have suggested that Hispanics may have more visceral fat, while Blacks may have less, compared to Whites [9, 10]; levels vary among Asian subgroups. For instance, Chinese and Japanese may have similar visceral fat [11–13], whereas Filipinos and South Asians exhibit greater visceral fat compared to Whites [12, 14]. However, it remains unknown to what degree visceral fat could explain racial differences in T2D. In the Women’s Health Initiative (WHI) Study, Black females had higher waist circumference than White females and the difference explained about 19% of higher risk of T2D [15]. Although a valuable measurement, waist circumference includes both visceral and subcutaneous fat around waist [8]. In addition, there may be sex differences in visceral fat [16–18] and sex and race interactions for visceral fat [19–23]. Thus, we aimed to examine whether visceral fat is a mediator and to what degree associations of race and ethnicity with incident T2D might be explained by visceral fat in sex subgroups based on data from a cohort study including multiple racial and ethnic groups.

## Materials and methods

### Study design and population

The Multi-Ethnic Study of Atherosclerosis (MESA) is a prospective cohort study including 6,814 adults aged 45–84 years, free of cardiovascular disease (CVD) at baseline. Participants were recruited from six US field centers between 2000 and 2002 (exam 1), followed by five subsequent examinations from 2002 to 2018, and 21 telephone follow-ups from 2001 to 2020 [24]. The current analysis included participants from an ancillary study, which included a 30% random sample (*n* = 1,947) of MESA cohort receiving abdominal computed tomography (CT) scans at either exam 2 (2002–2004) or exam 3 (2004–2005) [25, 26]. After excluding participants having T2D (*n* = 326) or with missing T2D status (*n* = 138) before or at visceral fat measurement, or those with missing visceral fat (*n* = 26), final sample included 1,457 participants (Supplementary Fig. S1). The study was approved by institutional review boards at all MESA sites, and participants provided written informed consent. Analysis period was June–November 2023, adhering to STROBE guidelines.

### Assessment of race and ethnicity

Race and ethnicity was self-reported as non-Hispanic White (White hereafter), Hispanic, non-Hispanic Black (Black hereafter), or non-Hispanic Chinese (Chinese hereafter) at exam 1.

### Assessment of visceral fat

Abdominal CT scans were obtained at exam 2 (*n* = 563) or exam 3 (*n* = 894). Two analysts used Medical Imaging Processing Analysis and Visualization 4.1.2 Software to assess visceral fat from six transverse slices at L2/L3, L3/L4, and L4/L5 vertebrae levels. Visceral fat was defined as adipose tissue enclosed by visceral cavity. We assessed visceral fat using the average of the six slices, with inter- and intra-rater reliability of 0.99 [27, 28].

### Assessment of type 2 diabetes

T2D was defined by (1) fasting plasma glucose ≥7.0 mmol/L at each exam or (2) self-reported use of glucose-lowering medications at each exam, or (3) self-reported diabetes diagnosis by physicians at exam 1, 5, 6 and at 21 telephone interviews.

### Assessment of covariates

Sociodemographic information (i.e., sex, marital status, education, income), chronic stress, medication use (i.e., hypertension, lipid-lowering), and smoking were collected at exam 1 from self-administrated questionnaires [24, 29]. We used age at measurement when visceral fat was scanned (i.e., exam 2 or 3) and we used age as the time scale in Cox proportional hazard regressions [30]. Chronic stress was assessed using Chronic Burden Scale at exam 1, categorized as low (score of 0), medium (score of 1), and high (score ≥2) [31]. Dietary intake was obtained using 120-item food frequency questionnaire (FFQ) at exam 1. Alternative Healthy Eating Index (AHEI)-2010 was calculated to indicate dietary quality [32]. Sedentary behavior [metabolic equivalent of task (MET)-hours/day] and exercise [MET-hours/day] were measured from Typical Week Physical Activity Survey (TWPAS) at exam 1 [24].

All anthropometric measures were directly collected by trained staff using standardized protocols [24]. Weight and height were assessed at exam 1 using a balance-beam scale and stadiometer, respectively. Body mass index (BMI) (kg/m^2^) was calculated as [weight (pound)*0.45]/[height (cm)/100]. Total body fat percentage at exam 1 was estimated using a validated anthropometric equation that shows strong agreement with dual-energy X-ray absorptiometry (DXA)-derived body fat [33]. In MESA, repeated examinations demonstrated excellent reliability of estimated fat mass, with an intraclass correlation coefficient of 0.93. We calculated total fat mass (kg) by weight* (total fat mass percentage/100). Abdominal muscle area (cm^2^) and density (Hounsfield units, HU) were assessed using CT scans. Resting blood pressure (BP) was assessed using an automated oscillometric sphygmomanometer at exam 1. Lipid profiles were assessed at exam 1 from plasma samples. Serum glucose was measured using a glucose oxidase method on the Vitros analyzer, and insulin by radioimmunoassay (Linco Human Insulin Specific RIA Kit). Homeostasis model assessment of insulin resistance (HOMA-IR) was calculated as fasting insulin (µU/mL) × fasting glucose (mmol/L) / 22.5, and homeostasis model assessment of β-cell function (HOMA-β) as 20 × fasting insulin (µU/mL) / (fasting glucose (mmol/L) − 3.5) [34].

### Statistical analysis

This study used SAS version 9.4 (SAS Institute, Cary, NC, USA) and R version 4.2.1. for analyses and considered statistically significant level as *P* < 0.05.

This study first assessed associations between race and T2D using Cox proportional hazards regressions, stratified by sex. The proportional hazards assumption was verified using Schoenfeld residuals. Adjusted models controlled for confounders of age and family history of diabetes. To minimize immortal time survival bias, age as the time scale with left truncation of age at study entry was used in Cox regressions [30]. Second, crude and age- and family history–adjusted distributions of visceral fat by race were examined across sex groups. In exploratory analysis, we examined mean visceral fat levels across BMI categories within each racial/ethnic group to explore potential evidence of a ‘thin–fat’ phenotype [35]. Third, this study assessed associations between visceral fat and T2D using Cox proportional hazards regressions, adjusting for age, family history of diabetes, marital status, education, income, stress, hypertension medication use, lipid-lowering medication use, smoking, AHEI-2010, sedentary behavior, exercise, BMI, total fat, muscle area and density, SBP, and lipids within each racial and sex subgroup. In additional exploratory analyses, we evaluated associations of visceral fat with HOMA-IR and HOMA-β using linear regression within each racial/ethnic group and tested visceral fat and race interactions using Wald χ² statistics to assess whether these relationships differed across groups.

If a racial minority group exhibited both a higher risk of T2D and greater visceral fat compared to White participants, and if visceral fat was positively associated with T2D within that group, we assessed the extent to which visceral fat mediated the association. Specifically, we quantified the extent to which differences in visceral fat explained racial disparities in T2D risk by estimating the mediation proportion using natural mediation effects [36] (Supplementary Fig. S2), with bootstrapping 200 times for 95% CIs. Total effect (TE, HR_TE_) was decomposed into natural indirect effect (NIE) (HR_Visceralfat_, red color in Supplementary Fig. S2) and natural direct effect (NDE) (HR_NotVisceralfat_, yellow color in Supplementary Fig. S2). Mediated proportion explained by visceral fat (cm^2^) was calculated from ln(HR_Visceralfat_)/ln(HR_TE_) [37].

Since missing percentages of each adjusted covariate were < 4.0%, completed data analyses in primary analysis were used. We conducted several sensitivity analyses to assess the robustness of our findings. First, we added the interaction between race and visceral fat on T2D in the mediation analysis. Second, used interventional path-specific effects (iPSEs) for mediation analysis (Supplementary Fig. S3) [38, 39]. More detailed procedures of iPSEs were in the Supplementary. Third, we replaced BMI with waist circumference and, separately, waist-to-hip ratio in the adjustment set. Fourth, we re-specified BMI using Asian BMI categories ( < 23, 23.0–27.4, ≥27.5 kg/m²; underweight <18.5 combined with normal weight due to very small numbers) and, in parallel, World Health Organization (WHO) categories ( < 25, 25.0–29.9, ≥30 kg/m², again combining underweight with normal weight) [40]. Fifth, we additionally adjusted for first-generation status (US/ Puerto Rico-born vs. foreign-born).

## Results

### Characteristics of study participants

Among 1457 participants, 51.2% were females; 44.0%, 23.0%, 19.4%, and 13.7% were White, Hispanic, Black, and Chinese, respectively. Hispanic and Black participants had higher percentages of having family history of T2D, had lower AHEI-2010 and higher BMI (kg/m^2^) than White participants. Hispanic, Black, and Chinese participants were less likely to have bachelor’s or higher degree and annual household income ≥$50,000 than White participants. All *P* values < 0.05 (Table 1).

**Table 1:** Characteristics of participants by racial and ethnic groups in Multi-Ethnic Study of Atherosclerosis (MESA) ancillary study.

Characteristics	Overall (*n* = 1457)		Whites [44.0% (641)]		Hispanics [23.0% (335)]			Blacks [19.4% (282)]			Chinese [13.7% (199)]		
							*P* values			*P* values			*P* values
Age (years)	64.58	(9.73)	65.34	(9.56)	62.93	(9.50)	<0.001	64.72	(10.01)	0.37	64.69	(10.04)	0.41
Female, % (*N*)	51.2	(746)	49.1	(315)	51.9	(174)	0.41	56.7	(160)	0.03	48.7	(97)	0.92
Family history of diabetes, % (*N*)	33.1	(474)	27.3	(174)	43.0	(142)	<0.001	43.0	(117)	<0.001	21.0	(41)	0.08
Married/living with a partner, % (*N*)	64.9	(936)	70.1	(448)	60.1	(196)	0.002	46.1	(129)	<0.001	82.3	(163)	0.001
Education, % (*N*)							<0.001			0.001			<0.001
High school or less	32.2	(468)	19.5	(125)	60.6	(203)		24.5	(69)		35.9	(71)	
Associates	28.9	(420)	29.2	(187)	24.8	(83)		37.2	(105)		22.7	(45)	
Bachelor’s or higher	39.0	(567)	51.3	(328)	14.6	(49)		38.3	(108)		41.4	(82)	
Annual household income, % (*N*)							<0.001			<0.001			<0.001
<$25,000	27.5	(389)	13.7	(86)	42.9	(141)		25.1	(66)		48.7	(96)	
$25,000–$49,999	28.6	(404)	25.6	(160)	33.4	(110)		34.2	(90)		22.3	(44)	
≥$50,000	44.0	(622)	60.7	(380)	23.7	(78)		40.7	(107)		28.9	(57)	
Stress, % (*N*)							0.37			0.11			<0.001
Low	42.1	(613)	37.2	(238)	41.8	(140)		39.7	(112)		61.8	(123)	
Middle	29.0	(422)	32.7	(209)	30.2	(101)		25.9	(73)		19.6	(39)	
High	28.9	(421)	30.2	(193)	28.1	(94)		34.4	(97)		18.6	(37)	
Hypertension medication use, % (*N*)	32.1	(468)	31.1	(199)	28.7	(96)	0.43	44.7	(126)	<0.001	23.6	(47)	0.04
Lipid-lowering medication use, % (*N*)	14.9	(217)	17.7	(113)	14.0	(47)	0.14	10.4	(29)	0.01	14.1	(28)	0.23
Cigarette smoking, % (*N*)							0.21			0.04			<0.001
Never	50.4	(733)	44.8	(287)	50.8	(170)		45.0	(127)		75.3	(149)	
Former	37.1	(540)	42.3	(271)	37.9	(127)		36.2	(102)		20.2	(40)	
Current	12.5	(182)	12.8	(82)	11.3	(38)		18.8	(53)		4.6	(9)	
Alternative healthy eating index (AHEI)-2010	53.40	(9.64)	54.43	(9.98)	50.85	(9.06)	<0.001	52.61	(10.00)	0.01	55.43	(7.89)	0.15
Sedentary behavior (MET- hours/day)	3.96	(2.62)	4.05	(2.70)	3.49	(2.31)	0.001	4.67	(2.83)	0.002	3.42	(2.31)	0.001
Exercise (MET-hour/day)	3.61	(4.12)	3.82	(4.02)	3.09	(3.54)	0.004	4.05	(5.26)	0.53	3.17	(3.33)	0.02
Body mass index (BMI, kg/m^2^)	27.34	(4.81)	27.07	(4.55)	28.70	(4.64)	<0.001	28.83	(5.24)	<0.001	23.77	(2.92)	<0.001
Total fat mass (kg)	26.83	(9.07)	26.97	(8.49)	28.17	(8.35)	0.04	29.87	(10.48)	<0.001	19.86	(5.85)	<0.001
Abdominal muscle area (cm^2^)	103.59	(28.89)	103.50	(28.39)	102.70	(29.10)	0.70	111.70	(30.52)	<0.001	93.93	(24.41)	<0.001
Abdominal muscle density (HU)	42.88	(5.27)	42.15	(5.24)	42.43	(5.15)	0.43	44.36	(5.46)	<0.001	43.87	(4.76)	<0.001
Systolic blood pressure (SBP, mmHg)	125.87	(21.33)	124.20	(20.22)	126.00	(22.64)	0.23	131.50	(21.47)	<0.001	123.10	(21.09)	0.51
Diastolic blood pressure (DBP, mmHg)	72.25	(10.04)	71.30	(10.02)	72.39	(9.97)	0.10	74.74	(10.32)	<0.001	71.57	(9.26)	0.74
Total cholesterol (mmol/L)	5.07	(0.88)	5.07	(0.89)	5.21	(0.92)	0.02	4.97	(0.87)	0.08	5.00	(0.77)	0.25
High-density lipoprotein (HDL) cholesterol (mmol/L)	1.35	(0.39)	1.37	(0.41)	1.26	(0.34)	<0.001	1.43	(0.41)	0.03	1.33	(0.33)	0.15
Low-density lipoprotein (LDL) cholesterol (mmol/L)	3.07	(0.78)	3.02	(0.75)	3.22	(0.83)	<0.001	3.06	(0.79)	0.50	3.02	(0.72)	0.94
Triglycerides (mmol/L)	1.43	(0.81)	1.5	(0.88)	1.62	(0.84)	0.05	1.02	(0.44)	<0.001	1.44	(0.76)	0.33

Data were presented as mean (standard deviation, SD) for continuous variables, and percentage, % (frequency, *N*) for categorical variables. Comparing each racial and ethnic minority group to White group, *P* values were compared using *t*-test for continuous variables and *χ*^2^-tests for categorical variables.

### Risk of type 2 diabetes by race and ethnicity

There were 294 participants developing incident T2D (incidence rate 17.0 per 1000 person-years) during a median follow-up time of 14.2 years. Among females, Hispanics had the highest crude incidence rate of T2D, followed by Chinese, Blacks, and Whites. Among males, Hispanics had the highest crude incidence rate of T2D, followed by Blacks, Whites, and Chinese. After adjusting for age and family history, in females, Hispanics (HR = 1.77, 95% CI: 1.17–2.69), Chinese [1.91 (1.15–3.15)], and Blacks [1.59 (1.02–2.49)] all had higher T2D risk than Whites. Compared to White males, Hispanic males [1.82 (1.20–2.76)] had higher T2D risk after adjusting for age and family history; Black males [1.48 (0.92–2.38)] also had a higher, non-statistically significant T2D risk, but Chinese males [0.86 (0.48–1.55)] had a lower, non-significant T2D risk (Table 2).

**Table 2:** Associations of race and ethnicity with type 2 diabetes (T2D), and associations of visceral fat with T2D.

Race and ethnicity and T2D																
	Females															
	Hispanic (*n* = 174) vs. White (*n* = 315)				Chinese (*n* = 97) vs. White (*n* = 315)				Black (*n* = 160) vs. White (*n* = 315)							
	HR	95%CI		*P*	HR	95%CI		*P*	HR	95%CI		*P*				
Unadjusted	**1.95**	**1.29**	**2.94**	**0.002**	**1.81**	**1.10**	**2.97**	**0.02**	**1.63**	**1.04**	**2.53**	**0.03**				
Adjusted for age and family history	**1.77**	**1.17**	**2.69**	**0.01**	**1.91**	**1.15**	**3.15**	**0.01**	**1.59**	**1.02**	**2.49**	**0.04**				
	**Males**															
	**Hispanic** **(*n*** = **161) vs. White (*n*** = **326)**				**Chinese (*n*** = **102) vs. White (*n*** = **326)**				**Black (*n*** = **122) vs. White (*n*** = **326)**							
	**HR**	**95%CI**		***P***	**HR**	**95%CI**		***P***	**HR**	**95%CI**		***P***				
Unadjusted	**2.04**	**1.36**	**3.04**	**0.001**	0.85	0.47	1.53	0.58	**1.74**	**1.11**	**2.73**	**0.02**				
Adjusted for age and family history	**1.82**	**1.20**	**2.76**	**0.005**	0.86	0.48	1.55	0.62	1.48	0.92	2.38	0.10				
**Visceral fat (10** **cm**^**2**^**) and T2D**																
	**Females**															
	**White (*n*** = **315)**				**Chinese (*n*** = **97)**				**Black (*n*** = **122)**				**Hispanic (*n*** = **161)**			
	**HR**	**95%CI**		***P***	**HR**	**95%CI**		***P***	**HR**	**95%CI**		***P***	**HR**	**95%CI**		***P***
Unadjusted	**1.12**	**1.07**	**1.16**	**<0.001**	**1.11**	**1.02**	**1.20**	**0.01**	**1.10**	**1.05**	**1.16**	**<0.001**	**1.09**	**1.04**	**1.14**	**0.001**
Multivariable adjusted	**1.11**	**1.02**	**1.20**	**0.01**	1.07	0.88	1.29	0.52	**1.31**	**1.12**	**1.53**	**0.001**	**1.19**	**1.08**	**1.33**	**0.001**
	**Males**															
	**White (*n*** = **315)**				**Chinese (*n*** = **97)**				**Black (*n*** = **122)**				**Hispanic (*n*** = **161)**			
	**HR**	**95%CI**		***P***	**HR**	**95%CI**		***P***	**HR**	**95%CI**		***P***	**HR**	**95%CI**		***P***
Unadjusted	**1.06**	**1.03**	**1.10**	**<0.001**	1.07	0.97	1.17	0.17	1.03	0.98	1.07	0.29	**1.06**	**1.02**	**1.10**	**0.004**
Multivariable adjusted	1.01	0.96	1.07	0.62	1.60	0.95	2.69	0.08	1.05	0.95	1.16	0.33	**1.10**	**1.03**	**1.19**	**0.01**

For race and T2D, visceral fat and T2D, cox proportional hazard regression models were used. Hazard ratio (HR), 95% confidence interval (CI) and *P* value were reported.

Boldface indicated statistical significance (i.e., *P* < 0.05).

For visceral fat and T2D, multivariable model adjusted for age, family history of diabetes, marital status, education, annual household income, stress, hypertension medication use, lipid-lowering medication use, smoking, alternative healthy eating index (AHEI)-2010, sedentary behavior, exercise, body mass index (BMI), total fat, muscle area and density, systolic blood pressure (SBP), total cholesterol, high-density lipoprotein (HDL) cholesterol, and triglycerides.

### Level of visceral fat by race and ethnicity

**Fig. 1:**
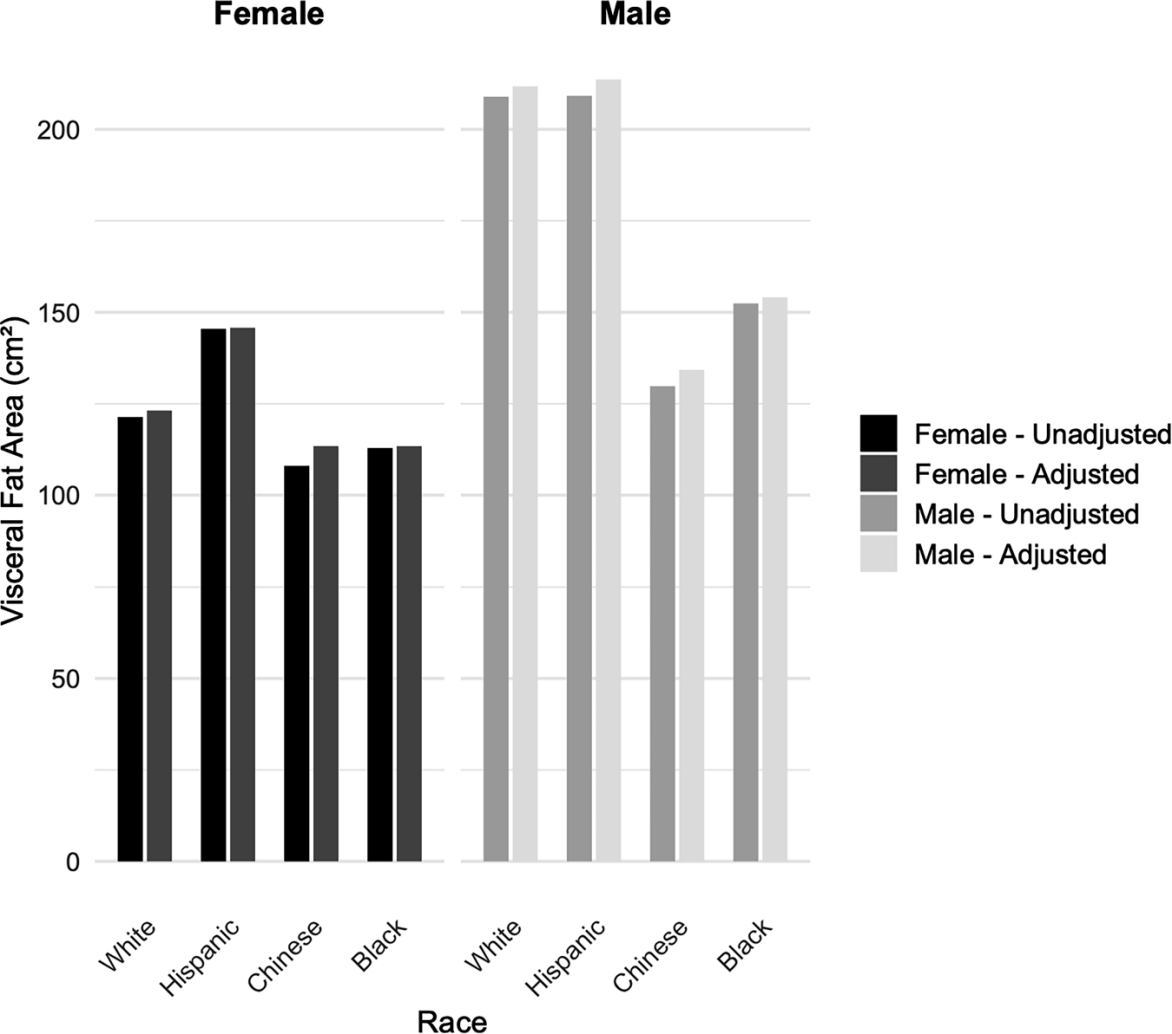
Visceral fat by racial and ethnic groups and sex groups in the Multi-Ethnic Study of Atherosclerosis (MESA) ancillary study. Unadjusted: crude mean of visceral fat. Adjusted: mean of visceral fat adjusted for age and family history of diabetes.

Among females, Hispanics had the highest visceral fat (cm^2^), followed by Whites, Blacks, and Chinese (Fig. 1). The difference in visceral fat, as compared to Whites still existing even after we adjusted for age and family history for Hispanic [mean difference = 22.72 (standard error, SE = 5.68), *P* < 0.001], but not for Chinese females [−9.44 (7.00), *P* = 0.18] and Black females [−9.71 (5.86), *P* = 0.10]. Among males, Hispanics and Whites had similar visceral fat, followed by Blacks and Chinese. Compared to White males, Hispanic males [1.66 (7.34), *P* = 0.38] had similar visceral fat (cm^2^), and Chinese males [−77.56 (8.47), *P* < 0.001] and Black males [−57.57 (8.11), *P* < 0.001] had lower visceral fat (cm^2^) even after adjusting for age and family history (Fig. 1).

In exploratory analyses, we examined visceral fat across BMI categories within each racial/ethnic group (Supplementary Table 1). Visceral fat increased with BMI in all groups, with little evidence of a classic ‘thin–fat’ phenotype [35] in this cohort.

### Associations of visceral fat with type 2 diabetes

In unadjusted models, a 10 cm^2^ higher visceral fat level was associated with 9% –12% higher risks of T2D in females [HR of 1.12 (95% CI: 1.07–1.16) for Whites, 1.11 (1.02–1.20) for Chinese, 1.10 (1.05–1.16) for Blacks, and 1.09 (1.04–1.14) for Hispanics], and 3% – 7% higher risks of T2D in males [HR of 1.06 (1.03–1.10)] for Whites, 1.07 (0.97–1.17) for Chinese, 1.03 (0.98–1.07) for Blacks, and 1.06 (1.02–1.10) for Hispanics]. After adjusting for age, family history of diabetes, marital status, education, annual household income, stress, hypertension medication use, lipid-lowering medication use, smoking, AHEI-2010, sedentary behavior, exercise, BMI, total fat, muscle area and density, SBP, total cholesterol, HDL cholesterol, and triglycerides, in females, visceral fat (10 cm^2^) was still positively associated with T2D in Whites [1.11 (1.02–1.20)], Blacks [1.31 (1.12–1.53)], Hispanics [1.19 (1.08–1.33)], but not in Chinese [1.07 (0.88–1.29)]. In males, visceral fat (10 cm^2^) was also positively associated with T2D in Hispanics [1.10 (1.03–1.19)], but not in Whites [1.01 (0.96–1.07)], Chinese [1.60 (0.95–2.69)], or Blacks [1.05 (0.95–1.16)] (Table 2).

In exploratory analyses, we examined race/ethnicity stratified associations of visceral adipose tissue with HOMA-IR and HOMA-β (Supplementary Table 2). Visceral fat was positively associated with HOMA-IR in White, Chinese, and Hispanic participants, with the strongest association in Hispanics; no significant association was observed among Black participants. The visceral fat and race interaction for HOMA-IR was statistically significant (*p*-interaction = 0.01), indicating heterogeneity in the strength of associations across racial/ethnic groups. For HOMA-β, point estimates were positive in all groups and statistically significant only in White and Hispanic participants. Associations were weaker and non-significant in Chinese and Black participants, and the visceral fat and race interaction for HOMA-β was not significant (*p*-interaction = 0.35), so these differences are considered exploratory.

### Mediation analyses of race and ethnicity and type 2 diabetes explained by visceral fat

**Fig. 2:**
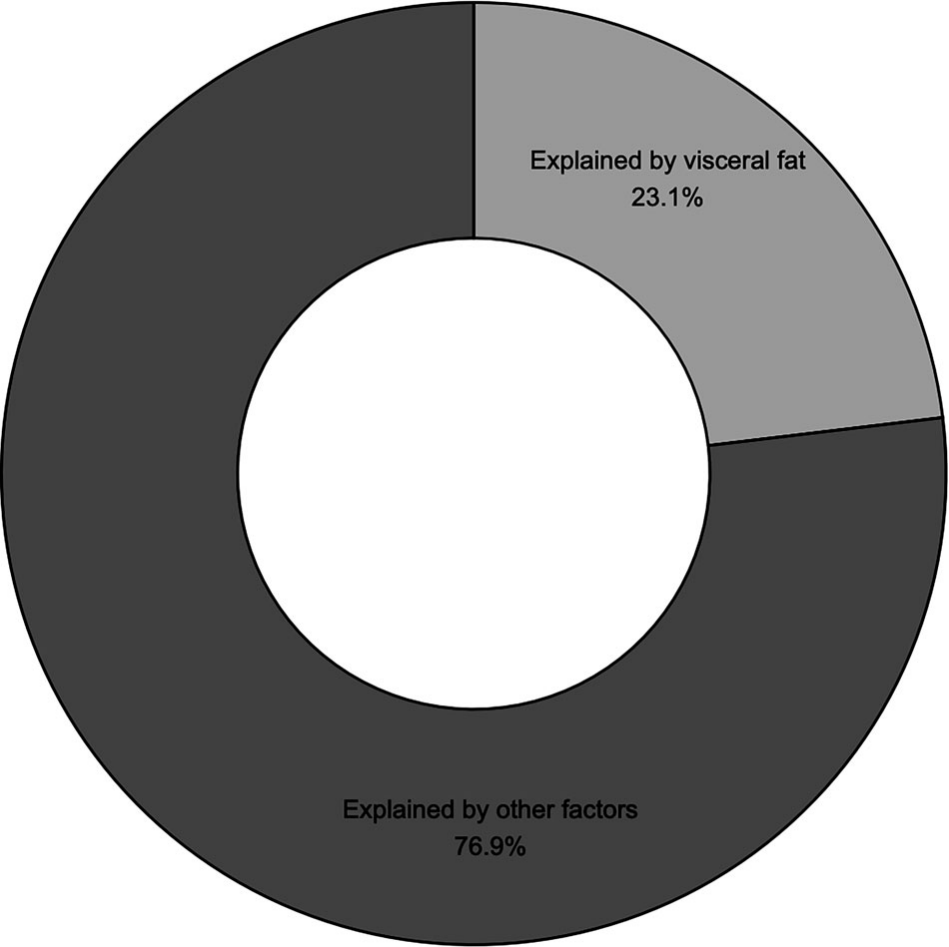
Decomposition of associations of race and ethnicity with type 2 diabetes (T2D) by visceral fat in Hispanic female vs. White female. Natural mediation effects were used to assessed racial and ethnic differences (Hispanic female vs. White female) in T2D explained by visceral fat.

As only Hispanic females met all three criteria (i.e., higher T2D risk, greater visceral fat than White counterpart, and a positive association between visceral fat and T2D in the group), we assessed the extent to which visceral fat mediated the T2D risk difference between Hispanic and White females. Using natural mediation effects, visceral fat explained 23.1% of racial differences in T2D comparing Hispanic females to White females (Fig. 2, Supplementary Table 3).

All sensitivity analyses yielded results that were consistent with the primary findings. Mediation proportions were similar when adding interactions between race and visceral fat on T2D in mediation analysis (Supplementary Table 4) or using iPSEs for mediation analysis (Supplementary Table 5), when replacing BMI with waist circumference (Supplementary Table 6) or waist-to-hip ratio (Supplementary Table 7), when using categorical BMI (Supplementary Table 8), and when additionally adjusting for first-generation status (Supplementary Table 9).

## Discussion

In this longitudinal cohort study including racially diverse participants in the US, we found after controlling for confounders, Hispanic, Black and Chinese females, as well as Hispanic males had higher risks of T2D than White counterparts, but Black and Chinese males did not have significantly different T2D risk compared with White males. Only Hispanic females had significantly higher visceral fat than White females. In addition, visceral fat may be a mediator of Hispanic females’ higher T2D risk, but not for Chinese and Black females’ and Hispanic and Black males’ higher T2D risk. Visceral fat explained about one-fifth of racial differences in T2D comparing Hispanic females to White females.

Racial differences in T2D have been linked to various factors like SES, lifestyles, and BMI [3, 15]. Our analysis also revealed that, adjusting for these factors reduced T2D risk disparities comparing Hispanic females and males with White counterparts (Supplementary Table 10), which preliminarily suggested that these mediators may partially explain racial disparities in T2D risk. Conversely, T2D risk increased in Chinese females, Black females, and Black males relative to White counterparts, and there was positive estimate of T2D risk in Chinese males compared to White males, after controlling for these mediators (Supplementary Table 10). Chinese females’ increased T2D risk was attributed to adjustments for smoking, AHEI-2010, and BMI, and increase for Chinese males was linked to BMI, total fat and visceral fat. The rise in risk for Black females and males was due to visceral fat (Supplementary Table 10). Because Chinese females had lower percentages of current smoking, higher AHEI-2010, and lower BMI, and Chinese males had lower BMI and total fat (Supplementary Table 11); and Chinese males [mean difference: -17.43 (7.37)], Black females [-31.72 (4.60)] and males [-51.60 (6.27)] had lower visceral fat (cm^2^) at equivalent BMI and total fat than White counterparts, controlling for these factors may reduce protective effect associated with healthier lifestyles and lower obesity metrics. More studies are needed to quantify mediation proportion for each mediator.

Although visceral fat is a well-known leading risk factor for T2D, few studies have examined to what degree visceral fat as a mediator may explain racial disparities in T2D. One study in the US found that Filipina women had higher visceral fat than White women, and further adjusting for education, exercise, alcohol drinking, and visceral fat, Filipinos’ odds of T2D decreased compared to Whites [14]. However, as visceral fat and other factors (e.g., alcohol drinking) were adjusted simultaneously, it was unclear whether decreased T2D risk was due to visceral fat or other factors and it was unknown to which degree racial differences in T2D were explained by visceral fat [14]. Since our data only had Chinese, future studies including more Asian subgroups (e.g., Filipino and South Asians) are needed [11–14].

We found Hispanic females had higher visceral fat than White females, which was also observed in an earlier published study in the US [19]. We did not find that visceral fat was a mediator for other minorities having higher T2D risks than Whites. It is likely because Black and Chinese females, as well as Hispanic males had similar visceral fat, but Black males had lower visceral fat compared to White counterparts in this study (Fig. 1), which was also observed in previous studies [11, 19–23]. For example, research in the US showed that Hispanic males had similar visceral fat mass compared to White males (6.2 vs. 6.0 kg) [19]. Another study in the US reported that Black females had similar visceral fat volume compared to White females (1.72 vs. 1.69 liters), whereas Black males had lower visceral fat volume than White males (2.48 vs. 3.40 liters) [20]. Additionally, a study in Singapore noted that Chinese-Singaporean females had similar visceral fat volume as White females (2.13 vs. 2.11 liters) [11].

Visceral fat may have been a mediator for higher T2D risk among Hispanic females due to their higher visceral fat than White females. Visceral adipose tissue has a relative high lipolysis rate which would increase flux of free fatty acid (FFA) from visceral fat depots to liver, leading to hepatic insulin resistance and hepatic steatosis [7]. Furthermore, visceral fat accumulation increases secretion of adipocytokines involving in inflammation and acute-phase response and influences hormones secretion, which are in regulation of insulin resistance [8]. Thus, visceral fat increases both hepatic insulin resistance and peripheral insulin resistance, which are bedrocks of development of T2D.

Although CT is the gold-standard method to assess visceral fat, it is expensive and not readily scalable for routine clinical care or community-based screening. In contrast, simple anthropometric measures such as waist circumference and waist-to-hip ratio are widely used because they are inexpensive, easy to obtain, and correlate reasonably well with CT-measured visceral fat. In our cohort, waist circumference and waist-to-hip ratio were moderately correlated with CT-measured visceral fat (r = 0.68 and 0.64, respectively), consistent with prior research [41]. These measures are useful for initial screening and risk stratification, particularly in resource-limited settings. However, they remain imperfect proxies for visceral fat, as waist circumference reflects a mixture of visceral and subcutaneous abdominal fat rather than visceral fat alone [8], so they cannot fully substitute for direct imaging when precise quantification of visceral adiposity is required.

### Strengths and limitations

This is the first cohort study including four racial and ethnic groups, examining to what degree racial and ethnic differences in T2D are mediated by visceral fat in the US. In addition, we could ensure temporality of associations due to time sequence of race, visceral fat and T2D. Moreover, visceral fat was measured by CT, allowing us to provide more precise estimates of the body fat composition than using waist circumference. Furthermore, our findings for mediation effects by visceral fat were robust, as we got similar results using two rigorous mediation analysis methods. Nonetheless, some potential limitations of our study may exist. First, as this was an observational study, residual confounding is possible despite adjustment for multiple covariates. For example, family history of diabetes was self-reported and may be misclassified, but it was used only as an adjustment covariate and had very low missingness (1.6%), such misclassification would be expected mainly to result in incomplete control for this confounder (residual confounding) rather than substantially bias our main associations, and excluding family history from the models generated similar results (Supplementary Table 12). Second, we do not have 75-g oral glucose tolerance test in MESA study, so 2-hour plasma glucose values were unavailable for defining T2D. As a result, some T2D cases may have been under-ascertained, which would likely bias associations toward the null and may partly explain the lack of a clear T2D difference among Asians. These subgroup findings should therefore be interpreted with appropriate caution. Third, visceral fat was measured only once, limiting our ability to capture changes in visceral fat over time. Nevertheless, a single midlife visceral fat measurement remains clinically informative because it reflects visceral fat accumulation for the development of insulin resistance [7] and has been shown to be associated with T2D [5, 42]. Future multi-racial studies with repeated visceral fat assessments are needed to evaluate how changes in visceral fat over time contribute to T2D risk and racial/ethnic disparities. Fourth, as we only included Chinese in MESA, future studies including more Asian subgroups are suggested. Fifth, we excluded 25% participants having T2D, with missing T2D status before or at visceral fat measurement, or with missing visceral fat. They were more likely to be Blacks and Hispanics, have lower education attainment, lower income than the included study sample (Supplementary Table 13). These may potentially introduce selection bias. However, we addressed this by applying inverse probability weighting to mitigate potential selection bias, which yielded similar mediation results (Supplementary Table 14). Finally, relatively small sample size of CT measured adiposity ancillary study may constrain detecting associations between race and T2D, race and visceral fat, visceral fat and T2D or mediation effects in sex subgroups. More studies with larger sample sizes are warranted.

## Conclusions

In this prospective cohort study including racially diverse participants, visceral fat could explain about one-fifth of racial and ethnic differences in T2D comparing Hispanic females to White females. Observing the role of visceral fat in T2D disparities improves understanding of the biological factor at play and may better inform planning clinical and public health strategies for diabetes prevention. Future research should test culturally tailored strategies to reduce visceral fat in different racial and ethnic groups and evaluate whether changes in visceral fat mediate reductions in T2D risk.

## Supplementary information

Supplement

## Data Availability

The datasets can be accessed by a reasonable request to the MESA Publication and Presentations Committee (https://www.mesa-nhlbi.org). Code used is available from the corresponding author upon reasonable request.

## Code availability

Code used is available from the corresponding author upon reasonable request.
