# Supplementary material for: Racial and ethnic disparities in type 2 diabetes: does visceral fat play a role? Evidence from the MESA study
Source: Int J Obes (Lond). 2026 Apr 8;50(6):1352–9. doi: 10.1038/s41366-026-02079-2 (PMC13286983; doi:10.1038/s41366-026-02079-2)
Supplement: Supplementary file 1 — Supplement [file 41366_2026_2079_MOESM1_ESM.pdf]

Supplementary Material

Supplementary Fig. S1. Participants flow chart

Supplementary Fig. S2. Analytical framework for parallel mediators, analyzed using natural mediation effects

Supplementary Fig. S3. Analytical framework for sequential mediators, analyzed using interventional path specific effects

Supplementary Table 1. Visceral fat distribution by body mass index category in each racial/ethnic group

Supplementary Table 2. Associations of visceral fat with homeostatic model assessment of insulin resistance (HOMR-IR) and homeostatic model assessment of beta-cell function (HOMA-β) in each race

Supplementary Table 3. Decomposition of associations of race and ethnicity with type 2 diabetes (T2D) by visceral fat

Supplementary Table 4. Decomposition of associations of race and ethnicity with type 2 diabetes (T2D) by visceral fat, adding interactions between race and visceral fat on T2D

Supplementary Table 5. Decomposition of associations of race and ethnicity with T2D by visceral fat, using interventional path specific effects (iPSEs)

Supplementary Table 6. Decomposition of associations of race and ethnicity with type 2 diabetes (T2D) by visceral fat, adjusting for waist circumstance replacing for body mass index

Supplementary Table 7. Decomposition of associations of race and ethnicity with type 2 diabetes (T2D) by visceral fat, adjusting for waist-to-hip ratio replacing for body mass index

Supplementary Table 8. Decomposition of associations of race and ethnicity with type 2 diabetes (T2D) by visceral fat, adjusting for categorical body mass index

Supplementary Table 9. Decomposition of associations of race and ethnicity with type 2 diabetes (T2D) by visceral fat, additionally adjusting for first-generation status

Supplementary Table 10. Associations of race and ethnicity with type 2 diabetes adjusting for mediators stepwise

Supplementary Table 11. Characteristics of participants by race and sex in Multi-Ethnic Study of Atherosclerosis (MESA) ancillary study

Supplementary Table 12. Decomposition of associations of race and ethnicity with type 2 diabetes (T2D) by visceral fat, removing family history of diabetes from the covariate set

Supplementary Table 13. Characteristics among included and excluded participants

Supplementary Table 14. Decomposition of associations of race and ethnicity with type 2 diabetes (T2D) by visceral fat, applying inverse probability weighting

Supplementary File

The participants flow chart was in **Supplementary Fig. S1.**

Interventional path-specific effects

We hypothesized potential mediators were parallel and used natural mediation effects[1] in primary analysis (**Supplementary Fig. S2)**. In sensitivity analysis, we hypothesized potential mediators were sequential and used the interventional path-specific effects (iPSEs)[2,3] (**Supplementary Fig. S3**). These two mediation approaches have different assumptions and the iPSE is more flexible as the natural mediation analysis requires no exposure-induced mediator-outcome confounders[2] which may exist in racial disparities research[4,5].

The estimation of iPSEs was conducted via g-computation, coupled with bootstrapping (200 iterations) to construct 95% confidence intervals (CIs)[1–3]. For iPSEs, we estimated hazard ratios (HRs) using Cox proportional hazards regression. We decomposed the interventional total effect (iTE) into two iPSEs,: (1) the indirect pathway: iPSE (Visceralfat) ($HR_{ipseVAT}$, red color in **Supplementary Fig. S3**) for the path through visceral fat; (2) the direct pathway: iPSE (NotVisceralfat) ($HR_{ipseNotVAT}$, yellow color in **Supplementary Fig. S3**) for the path not through visceral fat. The mediated proportion explained by visceral fat was calculated from $\ln(HR_{ipseVAT})/\ln(HR_{iTE})$ in iPSEs[6].

Methods of the interventional path specific effects (iPSEs)

Step 1: We built models for other mediators besides visceral fat, the mediator visceral fat and outcome Y, fitted these models using observed data, and obtained empirical parameters (coefficients).

Step 2: We simulated other mediators besides visceral fat, the mediator visceral fat and outcome Y sequentially based on models and parameters from the step 1.

Step 3. We obtained the E(Y ($x_1 = 1$, M($x_2 = 1$))), E(Y ($x_1 = 1$, M($x_2 = 0$))), and E(Y ($x_1 = 0$, M($x_2 = 0$))) based on models and parameters from the previous step 1 and step 2. We calculated the HR_{iTE} , $HR_{ipseVAT}$, and $HR_{ipseNotVAT}$ based on formula (1). We repeated step 2-3 on 200 bootstrapped samples taken at random with replacement from the original data to get 95% confidence interval (CI). We generated mediated proportion of each path from each iPSE and iTE using ln (HR of $iPSE_{\text{each path}}$)/ln(HR of iTE).

$$\left.\begin{aligned} \text{iPSE (VAT): } HR_{ipseVAT} &= EXP\,[\varphi\,(1,1) - \varphi\,(1,0)] \\ \text{iPSE (NotVAT): } HR_{ipseNotVAT} &= EXP\,[\varphi\,(1,0) - \varphi\,(0,0)] \\ \text{iTE: } HR_{iTE} &= EXP\,[\varphi\,(1,1) - \varphi\,(0,0)] \end{aligned}\right\} \quad \text{Formula (1)}$$

where φ (x_1, x_2) is defined as the E(Y (x_1, M(x_2))). Here, we set the exposure (race and ethnicity) as x_1; the mediator (visceral fat) as M(x_2). Race and ethnicity=1 represented Hispanic if comparing Hispanic vs. White; represented Chinese if comparing Chinese vs. White; and represented Black if comparing Black vs. White. The product of iPSE (Visceralfat) and iPSE (NotVisceralfat) was the iTE, which was expressed in HR scale as the following equation: $HR_{iTE} = HR_{ipseVAT} * HR_{ipseNotVAT}$

Reference

1. Wang A, Arah OA. G-computation demonstration in causal mediation analysis. *Eur J Epidemiol*. 2015;30(10):1119-1127. doi:10.1007/s10654-015-0100-z

2. VanderWeele TJ, Vansteelandt S, Robins JM. Effect Decomposition in the Presence of an Exposure-Induced Mediator-Outcome Confounder: *Epidemiology*. 2014;25(2):300-306. doi:10.1097/EDE.0000000000000034

3. Tai AS, Huang YT, Yang HI, Lan LV, Lin SH. G-Computation to Causal Mediation Analysis With Sequential Multiple Mediators—Investigating the Vulnerable Time Window of HBV Activity for the Mechanism of HCV Induced Hepatocellular Carcinoma. *Front Public Health*. 2022;9:757942. doi:10.3389/fpubh.2021.757942

4. Naimi AI, Kaufman JS. Counterfactual Theory in Social Epidemiology: Reconciling Analysis and Action for the Social Determinants of Health. *Curr Epidemiol Rep*. 2015;2(1):52-60. doi:10.1007/s40471-014-0030-4

5. VanderWeele TJ, Robinson WR. On the Causal Interpretation of Race in Regressions Adjusting for Confounding and Mediating Variables: *Epidemiology*. 2014;25(4):473-484. doi:10.1097/EDE.0000000000000105

6. VanderWeele TJ, Vansteelandt S. Odds Ratios for Mediation Analysis for a Dichotomous Outcome. *American Journal of Epidemiology*. 2010;172(12):1339-1348. doi:10.1093/aje/kwq332

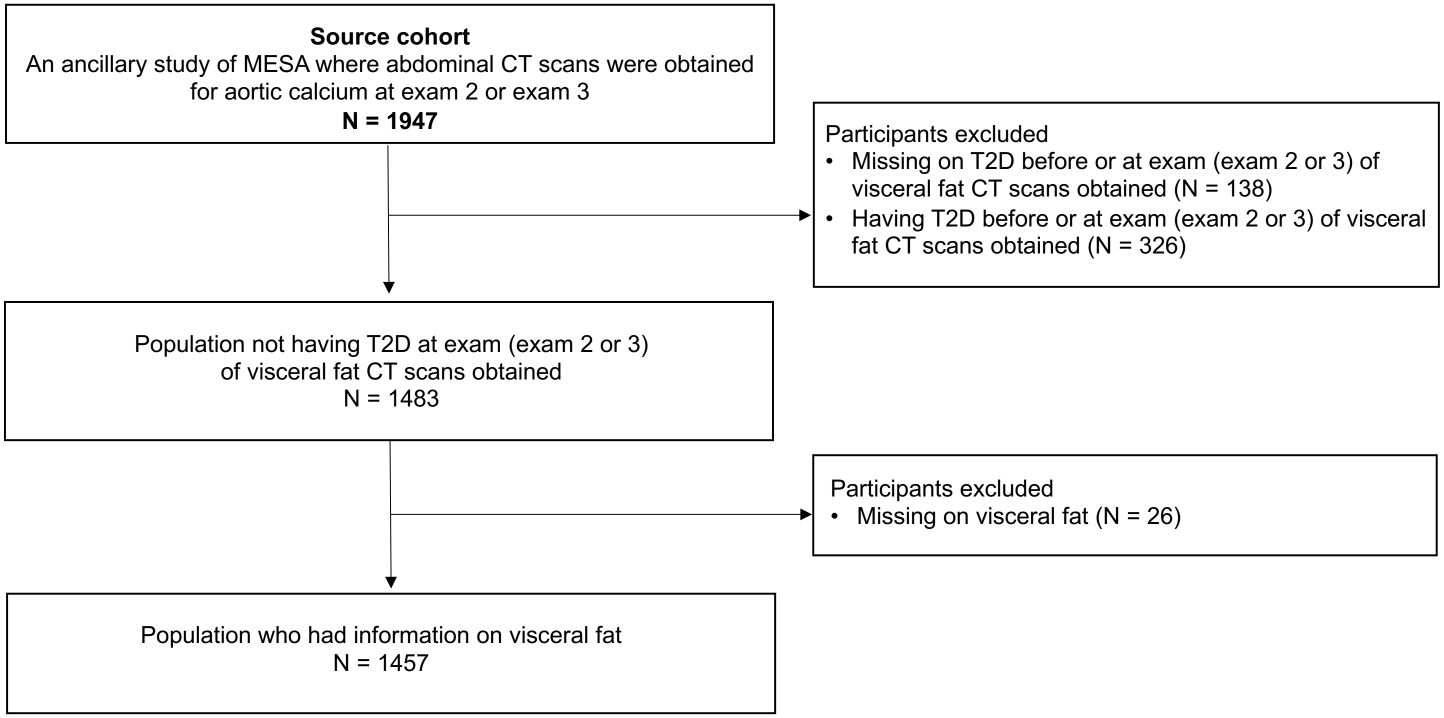

Supplementary Fig. S1. Participants flow chart

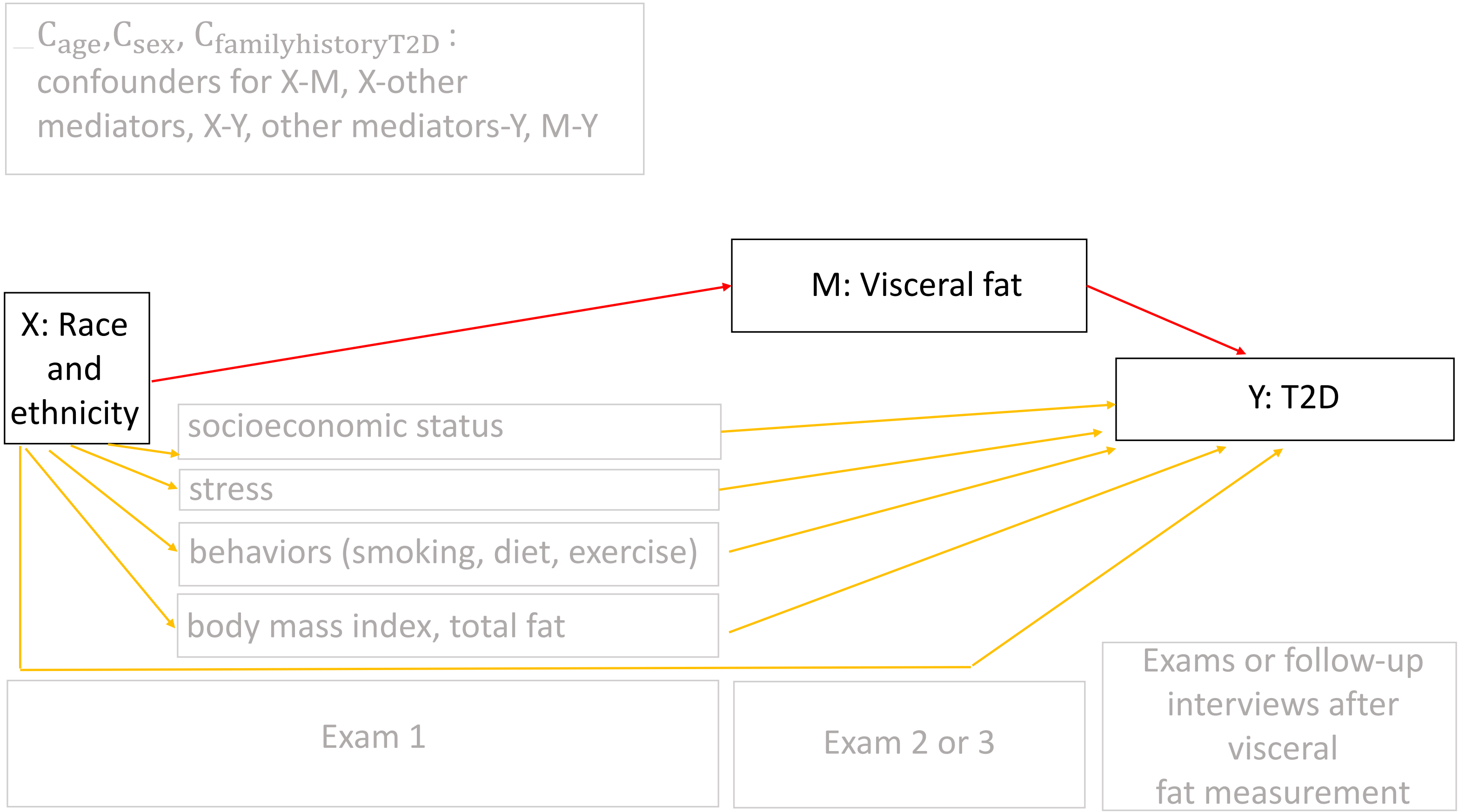

Supplementary Fig. S2. Analytical framework for parallel mediators, analyzed using natural mediation effects

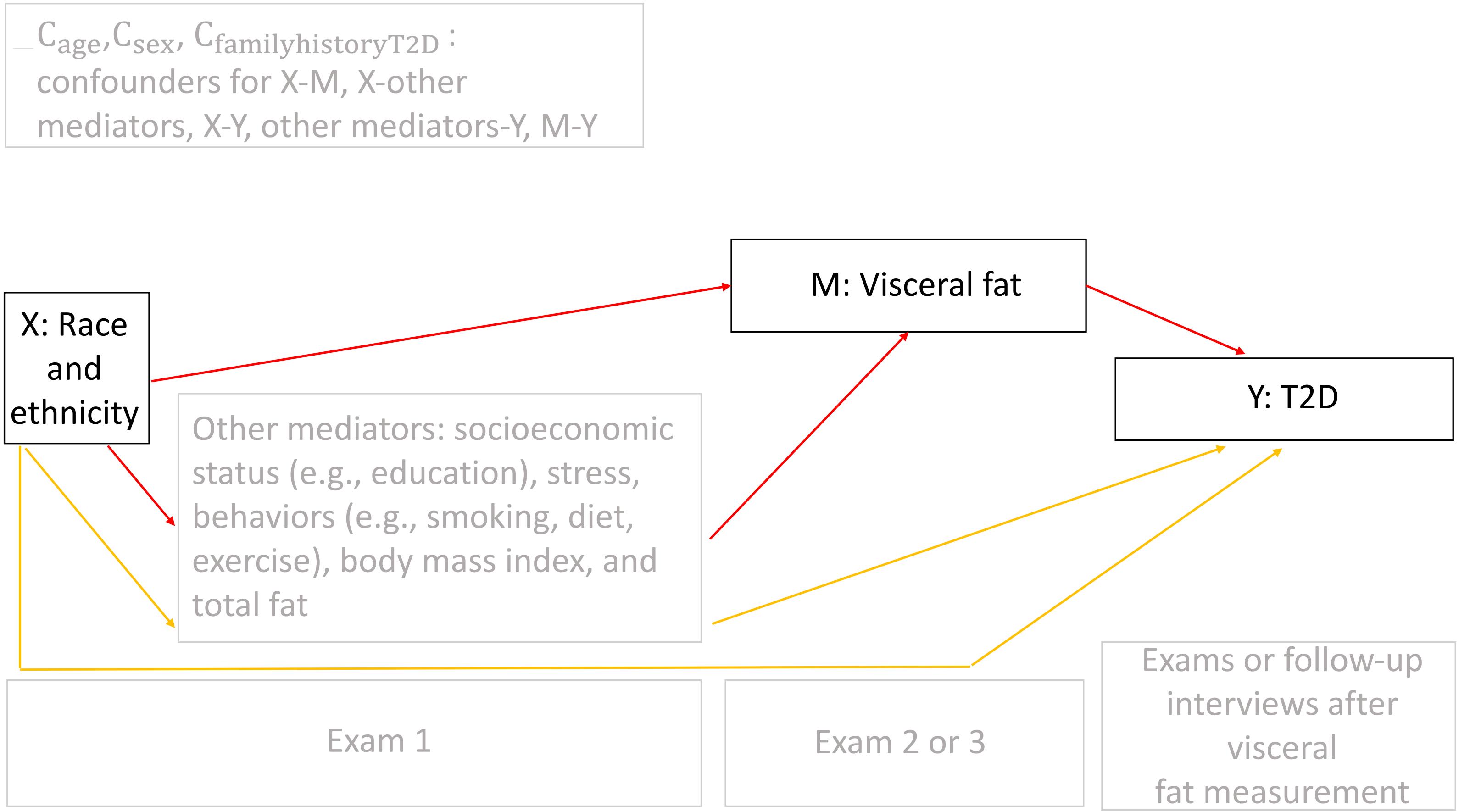

Supplementary Fig. S3. Analytical framework for sequential mediators, analyzed using interventional path specific effects

Supplementary Table 1. Visceral fat distribution by body mass index category in each racial/ethnic group

BMI categories	BMI <25 kg/m²	BMI 25–29.9 kg/m²	BMI ≥30 kg/m²
White	95.29 (51.96)	182.18 (72.20)	226.04 (77.97)
Chinese*	78.71 (31.75)	131.64 (44.01)	186.14 (44.64)
Black	87.56 (48.52)	119.09 (52.38)	170.47 (77.78)
Hispanic	116.31 (61.04)	167.56 (65.22)	214.69 (70.18)

Results were presented as mean (standard deviation).

*For Chinese participants, body mass index (BMI) categories used Asian cutpoints (<23.0, 23.0–27.4, ≥27.5 kg/m²; underweight <18.5 kg/m² was combined with normal weight due to small numbers). Whites, Blacks, and Hispanics used World Health Organization (WHO) cutpoints (<25.0, 25.0–29.9, ≥30 kg/m², again combining underweight with normal weight).

Supplementary Table 2. Associations of visceral fat with homeostatic model assessment of insulin resistance (HOMR-IR) and homeostatic model assessment of beta-cell function (HOMA-β) in each race

Race	Outcome	Estimate	95%CI		P
White	HOMA-IR	0.03	0.01	0.05	0.001
Chinese	HOMA-IR	0.04	0.01	0.07	0.01
Black	HOMA-IR	0.01	-0.02	0.04	0.61
Hispanic	HOMA-IR	0.07	0.04	0.10	<0.001
White	HOMA- β	0.69	0.07	1.31	0.03
Chinese	HOMA- β	0.68	-0.62	1.98	0.31
Black	HOMA- β	0.26	-1.06	1.58	0.70
Hispanic	HOMA- β	1.76	0.84	2.68	<0.001

Linear regression models were used.

Visceral adipose tissue was analyzed per cm^2 increment.

Models adjusted for age, gender, family history of diabetes, marital status, education, annual household income, stress, hypertension medication use, lipid-lowering medication use, smoking, alternative healthy eating index (AHEI)-2010, sedentary behavior, exercise, body mass index (BMI), total fat, muscle area and density, systolic blood pressure (SBP), total cholesterol, high-density lipoprotein (HDL) cholesterol, and triglyceride.

Supplementary Table 3. Decomposition of associations of race and ethnicity with type 2 diabetes (T2D) by visceral fat

	Natural indirect effect (NIE)					Natural direct effect (NDE)				Total effect (TE)			
	**HR**	**95%CI**		***P***	**Proportion explained (%)**	**HR**	**95%CI**		***P***	**HR**	**95%CI**		***P***
Female													
Hispanic vs. White	1.22	0.88	1.70	0.12	23.1%	**1.97**	**1.17**	**4.75**	**0.02**	**2.40**	**1.45**	**5.82**	**<0.001**
Chinese vs. White	0.91	0.72	1.15	0.46		**3.01**	**1.53**	**8.60**	**<0.001**	**2.78**	**1.43**	**7.52**	**<0.001**
Black vs. White	0.87	0.61	1.14	0.23		1.84	0.93	4.39	0.09	1.58	0.81	3.50	0.16
Male													
Hispanic vs. White	1.05	0.86	1.25	0.94		**2.15**	**1.25**	**4.16**	**0.01**	**2.18**	**1.26**	**4.20**	**0.01**
Chinese vs. White	0.95	0.46	1.25	0.42		1.02	0.32	3.93	0.88	0.88	0.26	2.46	0.78
Black vs. White	0.93	0.65	1.36	0.76		1.25	0.63	3.34	0.46	1.19	0.59	2.84	0.52

Natural mediation effects were used to assessed racial and ethnic differences in T2D explained by visceral fat. Bootstrap was used to get 95% confidence interval (CI) and P-values. HR, 95% CI and P-value were reported. Boldface indicated statistical significance (i.e., P<0.05).

Continuous visceral fat (cm^2) was used as the mediator.

Type 2 diabetes (T2D) was regressed on age, sex, family history of diabetes, race and ethnicity, marital status, education, annual household income, stress, hypertension medication use, lipid-lowering medication use, smoking, alternative healthy eating index (AHEI)-2010, sedentary behavior, exercise, body mass index (BMI), total fat mass, abdominal muscle area and density, systolic blood pressure (SBP), total cholesterol, high-density lipoprotein (HDL) cholesterol, triglycerides, and visceral fat.

Supplementary Table 4. Decomposition of associations of race and ethnicity with type 2 diabetes (T2D) by visceral fat, adding interactions between race and visceral fat on T2D

		**Natural indirect effect (NIE)**					**Natural direct effect (NDE)**				**Total effect (TE)**			
	**HR**	**95%CI**		***P***	**Proportion explained (%)**	**HR**	**95%CI**		***P***	**HR**	**95%CI**		***P***
Female													
Hispanic vs. White	1.23	0.90	1.78	0.14	24.2%	**1.93**	**1.06**	**4.46**	**0.03**	**2.36**	**1.42**	**6.04**	**<0.001**
Chinese vs. White	0.91	0.64	1.21	0.57		**3.00**	**1.25**	**7.38**	**0.02**	**2.76**	**1.34**	**6.67**	**0.01**
Black vs. White	0.88	0.59	1.14	0.35		1.84	0.92	4.13	0.08	1.58	0.80	3.53	0.16
Male													
Hispanic vs. White	1.05	0.86	1.29	0.91		**2.00**	**1.09**	**3.83**	**0.02**	**2.04**	**1.11**	**4.02**	**0.03**
Chinese vs. White	0.54	0.07	1.43	0.15		1.60	0.47	6.77	0.42	0.79	0.15	2.00	0.61
Black vs. White	1.05	0.70	1.85	0.71		1.20	0.54	3.06	0.59	1.29	0.71	3.33	0.38

Natural mediation effects were used to assessed racial and ethnic differences in T2D explained by visceral fat. Bootstrap was used to get 95% confidence interval (CI) and P-values. HR, 95% CI and P-value were reported. Boldface indicated statistical significance (i.e., $P<0.05$).
Continuous visceral fat (cm^2) was used as the mediator.
Interactions between race and visceral fat on T2D were added in the mediation analysis.
Type 2 diabetes (T2D) was regressed on age, sex, family history of diabetes, race and ethnicity, marital status, education, annual household income, stress, hypertension medication use, lipid-lowering medication use, smoking, alternative healthy eating index (AHEI)-2010, sedentary behavior, exercise, body mass index (BMI), total fat mass, abdominal muscle area and density, systolic blood pressure (SBP), total cholesterol, high-density lipoprotein (HDL) cholesterol, triglycerides, and visceral fat.

Supplementary Table 5. Decomposition of associations of race and ethnicity with T2D by visceral fat, using interventional path specific effects (iPSEs)

	iPSE (Visceralfat)					iPSE (notVisceralfat)				iTE			
	**HR**	**95%CI**		***P***	**Proportion explained (%)**	**HR**	**95%CI**		***P***	**HR**	**95%CI**		***P***
Female													
Hispanic vs. White	**1.20**	**1.04**	**1.54**	**<0.001**	20.3%	**2.08**	**1.30**	**3.90**	**0.01**	**2.50**	**1.50**	**5.43**	**0.01**
Chinese vs. White	0.92	0.77	1.03	0.20		**2.59**	**1.27**	**7.96**	**0.01**	**2.38**	**1.19**	**7.37**	**0.03**
Black vs. White	0.83	0.64	1.01	0.06		2.11	1.02	4.13	0.05	1.75	0.81	3.78	0.14
Male													
Hispanic vs. White	1.02	0.95	1.11	0.71		**1.95**	**1.23**	**4.42**	**<0.001**	**1.99**	**1.24**	**4.63**	**<0.001**
Chinese vs. White	0.91	0.55	1.29	0.54		0.99	0.32	2.98	0.98	0.91	0.30	2.11	0.80
Black vs. White	0.98	0.78	1.22	0.90		1.24	0.73	3.20	0.37	1.22	0.76	2.87	0.34

Interventional path specific effects (iPSEs) were used to assessed racial and ethnic differences in T2D explained by visceral fat. Hazard ratio (HR), 95% confidence interval (CI) and P-value were reported. Boldface indicated statistical significance (i.e., P<0.05).

Continuous visceral fat (cm^2) was used as the mediator.

Type 2 diabetes (T2D) was regressed on age, sex, family history of diabetes, race and ethnicity, marital status, education, annual household income, stress, hypertension medication use, lipid-lowering medication use, smoking, alternative healthy eating index (AHEI)-2010, sedentary behavior, exercise, body mass index (BMI), total fat mass, systolic blood pressure (SBP), total cholesterol, high-density lipoprotein (HDL) cholesterol, triglycerides, and visceral fat.

iPSE, interventional path specific effects; iTE, interventional total effect.

Supplementary Table 6. Decomposition of associations of race and ethnicity with type 2 diabetes (T2D) by visceral fat, adjusting for waist circumstance replacing for body mass index

	**Natural indirect effect (NIE)**					**Natural direct effect (NDE)**				**Total effect (TE)**			
	**HR**	**95%CI**		***P***	**Proportion explained (%)**	**HR**	**95%CI**		***P***	**HR**	**95%CI**		***P***
Female													
Hispanic vs. White	**1.26**	**1.10**	**1.36**	**<0.001**	23.2	**2.18**	**1.90**	**2.40**	**<0.001**	**2.66**	**2.31**	**2.92**	**<0.001**
Chinese vs. White	0.97	0.81	1.06	0.30		**3.17**	**2.57**	**3.32**	**<0.001**	**2.92**	**2.37**	**3.06**	**<0.001**
Black vs. White	0.94	0.77	1.00	0.06		**2.33**	**1.94**	**2.47**	**<0.001**	**2.04**	**1.69**	**2.16**	**<0.001**
Male													
Hispanic vs. White	1.09	0.91	1.12	0.74		**1.68**	**1.57**	**1.87**	**<0.001**	**1.71**	**1.59**	**1.90**	**<0.001**
Chinese vs. White	0.86	0.74	1.06	0.16		1.01	0.88	1.28	0.62	0.87	0.76	1.10	0.32
Black vs. White	0.88	0.79	1.14	0.44		**1.30**	**1.04**	**1.41**	**0.03**	1.20	0.96	1.30	0.15

Natural mediation effects were used to assessed racial and ethnic differences in T2D explained by visceral fat. Bootstrap was used to get 95% confidence interval (CI) and P-values. HR, 95% CI and P-value were reported. Boldface indicated statistical significance (i.e., $P<0.05$).

Continuous visceral fat (cm^2) was used as the mediator.

Type 2 diabetes (T2D) was regressed on age, sex, family history of diabetes, race and ethnicity, marital status, education, annual household income, stress, hypertension medication use, lipid-lowering medication use, smoking, alternative healthy eating index (AHEI)-2010, sedentary behavior, exercise, **waist circumstance,** total fat mass, abdominal muscle area and density, systolic blood pressure (SBP), total cholesterol, high-density lipoprotein (HDL) cholesterol, triglycerides, and visceral fat.

Supplementary Table 7. Decomposition of associations of race and ethnicity with type 2 diabetes (T2D) by visceral fat, adjusting for waist-to-hip ratio replacing for body mass index

	**Natural indirect effect (NIE)**					**Natural direct effect (NDE)**				**Total effect (TE)**			
	**HR**	**95%CI**		***P***	**Proportion explained (%)**	**HR**	**95%CI**		***P***	**HR**	**95%CI**		***P***
Female													
Hispanic vs. White	**1.26**	**1.10**	**1.35**	**<0.001**	23.6	**2.16**	**1.89**	**2.37**	**<0.001**	**2.62**	**2.29**	**2.88**	**<0.001**
Chinese vs. White	0.97	0.81	1.06	0.30		**3.16**	**2.57**	**3.32**	**<0.001**	**2.92**	**2.37**	**3.06**	**<0.001**
Black vs. White	0.94	0.78	1.00	0.05		**2.29**	**1.91**	**2.42**	**<0.001**	**2.00**	**1.68**	**2.12**	**<0.001**
Male													
Hispanic vs. White	1.09	0.92	1.12	0.72		**1.69**	**1.58**	**1.88**	**<0.001**	**1.71**	**1.60**	**1.91**	**<0.001**
Chinese vs. White	0.86	0.74	1.05	0.12		1.02	0.89	1.28	0.58	0.87	0.76	1.10	0.29
Black vs. White	0.89	0.79	1.15	0.46		**1.28**	**1.02**	**1.39**	**0.03**	1.18	0.94	1.28	0.22

Natural mediation effects were used to assessed racial and ethnic differences in T2D explained by visceral fat. Bootstrap was used to get 95% confidence interval (CI) and P-values. HR, 95% CI and P-value were reported. Boldface indicated statistical significance (i.e., P<0.05).

Continuous visceral fat (cm^2) was used as the mediator.

Type 2 diabetes (T2D) was regressed on age, sex, family history of diabetes, race and ethnicity, marital status, education, annual household income, stress, hypertension medication use, lipid-lowering medication use, smoking, alternative healthy eating index (AHEI)-2010, sedentary behavior, exercise, **waist-to-hip ratio,** total fat mass, abdominal muscle area and density, systolic blood pressure (SBP), total cholesterol, high-density lipoprotein (HDL) cholesterol, triglycerides, and visceral fat.

Supplementary Table 8. Decomposition of associations of race and ethnicity with type 2 diabetes (T2D) by visceral fat, adjusting for categorical body mass index

	Natural indirect effect (NIE)					Natural direct effect (NDE)				Total effect (TE)			
	**HR**	**95%CI**		***P***	**Proportion explained (%)**	**HR**	**95%CI**		***P***	**HR**	**95%CI**		***P***
Female													
Hispanic vs. White	**1.25**	**1.10**	**1.35**	**<0.001**	26.2	**1.96**	**1.90**	**2.36**	**<0.001**	**2.37**	**2.30**	**2.86**	**<0.001**
Chinese vs. White	0.79	0.81	1.05	0.26		**3.20**	**2.91**	**3.65**	**<0.001**	**2.94**	**2.67**	**3.36**	**<0.001**
Black vs. White	0.94	0.75	1.02	0.11		**2.21**	**1.97**	**2.64**	**<0.001**	**1.92**	**1.72**	**2.29**	**<0.001**
Male													
Hispanic vs. White	0.99	0.89	1.15	0.69		**1.81**	**1.53**	**2.00**	**<0.001**	**1.84**	**1.56**	**2.03**	**<0.001**
Chinese vs. White	0.95	0.71	1.04	0.12		1.09	0.86	1.26	0.49	0.93	0.74	1.08	0.29
Black vs. White	1.07	0.77	1.11	0.56		1.26	0.95	1.39	0.20	1.17	0.89	1.30	0.50

Natural mediation effects were used to assessed racial and ethnic differences in T2D explained by visceral fat. Bootstrap was used to get 95% confidence interval (CI) and P-values. HR, 95% CI and P-value were reported. Boldface indicated statistical significance (i.e., P<0.05).
Continuous visceral fat (cm^2) was used as the mediator.
Type 2 diabetes (T2D) was regressed on age, sex, family history of diabetes, race and ethnicity, marital status, education, annual household income, stress, hypertension medication use, lipid-lowering medication use, smoking, alternative healthy eating index (AHEI)-2010, sedentary behavior, exercise, **categorical body mass index,** total fat mass, abdominal muscle area and density, systolic blood pressure (SBP), total cholesterol, high-density lipoprotein (HDL) cholesterol, triglycerides, and visceral fat.

Supplementary Table 9. Decomposition of associations of race and ethnicity with type 2 diabetes (T2D) by visceral fat, additionally adjusting for first-generation status

	Natural indirect effect (NIE)					Natural direct effect (NDE)				Total effect (TE)			
	**HR**	**95%CI**		***P***	**Proportion explained (%)**	**HR**	**95%CI**		***P***	**HR**	**95%CI**		***P***
Female													
Hispanic vs. White	**1.13**	**1.08**	**1.36**	**<0.001**	24.5	**1.39**	**1.31**	**1.59**	**<0.001**	**1.67**	**1.57**	**1.92**	**<0.001**
Chinese vs. White	1.00	0.82	1.04	0.16		0.95	0.88	1.15	0.96	0.87	0.81	1.06	0.28
Black vs. White	1.00	0.68	1.11	0.23		**1.61**	**1.67**	**2.50**	**<0.001**	**1.40**	**1.45**	**2.17**	**<0.001**
Male													
Hispanic vs. White	0.91	0.89	1.15	0.75		1.08	0.88	1.10	0.77	1.09	0.89	1.12	0.87
Chinese vs. White	0.88	0.71	1.07	0.33		**0.20**	**0.14**	**0.23**	**<0.001**	**0.18**	**0.13**	**0.20**	**<0.001**
Black vs. White	0.91	0.78	1.10	0.54		0.99	0.87	1.26	0.63	0.94	0.82	1.19	0.85

Natural mediation effects were used to assessed racial and ethnic differences in T2D explained by visceral fat. Bootstrap was used to get 95% confidence interval (CI) and P-values. HR, 95% CI and P-value were reported. Boldface indicated statistical significance (i.e., $P<0.05$).

Continuous visceral fat (cm^2) was used as the mediator.

Type 2 diabetes (T2D) was regressed on age, sex, family history of diabetes, race and ethnicity, **first-generation status,** marital status, education, annual household income, stress, hypertension medication use, lipid-lowering medication use, smoking, alternative healthy eating index (AHEI)-2010, sedentary behavior, exercise, BMI, total fat mass, abdominal muscle area and density, systolic blood pressure (SBP), total cholesterol, high-density lipoprotein (HDL) cholesterol, triglycerides, and visceral fat.

Supplementary Table 10. Associations of race and ethnicity with type 2 diabetes adjusting for mediators stepwise

	Race/ethnicity and T2D											
	**Females**											
	**Hispanic vs. White**				**Chinese vs. White**				**Black vs. White**			
	**HR**	**95%CI**		**P**	**HR**	**95%CI**		**P**	**HR**	**95%CI**		**P**
Model 1	**1.95**	**1.29**	**2.94**	**0.002**	**1.81**	**1.10**	**2.97**	**0.02**	**1.63**	**1.04**	**2.53**	**0.03**
Model 2	**1.77**	**1.17**	**2.69**	**0.01**	**1.91**	**1.15**	**3.15**	**0.01**	1.59	1.02	2.49	0.04
Model 3	1.31	0.84	2.05	0.24	1.49	0.89	2.51	0.13	1.59	1.01	2.50	0.05
Model 4	1.35	0.86	2.11	0.20	1.49	0.88	2.51	0.14	1.61	1.01	2.55	0.05
Model 5	1.30	0.83	2.05	0.25	1.42	0.83	2.45	0.20	1.60	1.00	2.55	0.05
Model 6	1.37	0.87	2.15	0.18	1.56	0.90	2.69	0.11	1.59	0.99	2.56	0.05
Model 7	1.44	0.91	2.29	0.12	1.69	0.95	3.02	0.08	1.61	1.00	2.59	0.05
Model 8	1.37	0.86	2.21	0.19	1.85	1.03	3.35	0.04	1.56	0.96	2.55	0.08
Model 9	1.33	0.82	2.15	0.25	1.79	0.99	3.25	0.06	1.58	0.97	2.59	0.07
Model 10	1.21	0.75	1.96	0.44	2.00	1.09	3.64	0.02	1.37	0.83	2.26	0.22
Model 11	1.25	0.76	2.06	0.38	2.04	1.11	3.73	0.02	1.39	0.84	2.30	0.20
Model 12	1.31	0.79	2.16	0.30	2.27	1.21	4.29	0.01	1.25	0.72	2.19	0.43
Model 13	1.29	0.78	2.15	0.32	2.11	1.10	4.04	0.03	1.77	1.00	3.13	0.05
	**Males**											
	**Hispanic vs. White**				**Chinese vs. White**				**Black vs. White**			
	**HR**	**95%CI**		**P**	**HR**	**95%CI**		**P**	**HR**	**95%CI**		**P**
Model 1	**2.04**	**1.36**	**3.04**	**0.001**	0.85	0.47	1.53	0.58	**1.74**	**1.11**	**2.73**	**0.02**
Model 2	**1.82**	**1.20**	**2.76**	**0.005**	0.86	0.48	1.55	0.62	1.48	0.92	2.38	0.10
Model 3	1.31	0.82	2.09	0.25	0.72	0.38	1.36	0.31	1.33	0.81	2.18	0.27
Model 4	1.35	0.84	2.15	0.21	0.71	0.37	1.36	0.30	1.34	0.81	2.21	0.25
Model 5	1.33	0.83	2.13	0.23	0.70	0.36	1.35	0.29	1.34	0.81	2.20	0.26
Model 6	1.33	0.83	2.14	0.23	0.74	0.38	1.44	0.38	1.37	0.82	2.27	0.23
Model 7	1.34	0.83	2.14	0.23	0.75	0.38	1.46	0.39	1.37	0.82	2.28	0.23
Model 8	1.28	0.79	2.07	0.32	0.74	0.38	1.46	0.39	1.37	0.82	2.28	0.23
Model 9	1.37	0.84	2.22	0.21	0.77	0.40	1.50	0.44	1.40	0.84	2.35	0.20
Model 10	1.27	0.78	2.08	0.34	0.99	0.50	1.98	0.98	1.42	0.85	2.37	0.18

Model 11	1.46	0.88	2.42	0.15	1.16	0.57	2.35	0.68	1.44	0.86	2.41	0.16
Model 12	1.47	0.88	2.47	0.15	1.18	0.59	2.39	0.64	1.65	0.95	2.86	0.08
Model 13	1.53	0.91	2.59	0.11	1.40	0.68	2.87	0.36	1.98	1.12	3.50	0.02

Cox proportional hazard regression models were used. Hazard ratio (HR), 95% confidence interval (CI) and P-value were reported. Boldface indicated statistical significance after multiple testing [i.e., false-discovery rate (FDR) <0.05 using the Benjamini-Hochberg method].
Model 1: Crude model.
Model 2: Adjust for confounders, age and family history of diabetes.
From model 3, mediators were additionally adjusted.
Model 3: Adjust for age, gender, family history of diabetes, education, and annual household income.
Model 4: Adjust for age, gender, family history of diabetes, education, annual household income, and marital status.
Model 5: Adjust for age, gender, family history of diabetes, education, annual household income, marital status, and stress.
Model 6: Adjust for age, gender, family history of diabetes, education, annual household income, marital status, stress, and hypertension medication use, lipid-lowering medication use.
Model 7: Adjust for age, gender, family history of diabetes, education, annual household income, marital status, stress, hypertension medication use, lipid-lowering medication use, and smoking.
Model 8: Adjust for age, gender, family history of diabetes, education, annual household income, marital status, stress, hypertension medication use, lipid-lowering medication use, smoking, and AHEI-2010.
Model 9: Adjust for age, gender, family history of diabetes, education, annual household income, marital status, stress, hypertension medication use, lipid-lowering medication use, smoking, AHEI-2010, exercise, and sedentary behavior.
Model 10: Adjust for age, gender, family history of diabetes, education, annual household income, marital status, stress, hypertension medication use, lipid-lowering medication use, smoking, AHEI-2010, exercise, sedentary behavior, and BMI.
Model 11: Adjust for age, gender, family history of diabetes, education, annual household income, marital status, stress, hypertension medication use, lipid-lowering medication use, smoking, AHEI-2010, exercise, sedentary behavior, BMI, and total fat.
Model 12: Adjust for age, gender, family history of diabetes, education, annual household income, marital status, stress, hypertension medication use, lipid-lowering medication use, smoking, AHEI-2010, exercise, sedentary behavior, BMI, total fat, muscle area and density, SBP, total cholesterol, HDL cholesterol, and triglycerides.
Model 13: Adjust for age, gender, family history of diabetes, education, annual household income, marital status, stress, hypertension medication use, lipid-lowering medication use, smoking, AHEI-2010, exercise, sedentary behavior, BMI, total fat, muscle area and density, SBP, total cholesterol, HDL cholesterol, triglycerides, and visceral fat.
AHEI-2010, alternative healthy eating index-2010; BMI, body mass index; HDL cholesterol, high-density lipoprotein cholesterol; SBP, systolic blood pressure.

Supplementary Table 11. Characteristics of participants by race and sex in Multi-Ethnic Study of Atherosclerosis (MESA) ancillary study

Characteristics	Non-Hispanic White females [21.6% (315)]		Hispanic females [11.9% (174)]		P-values compared to White females	Non-Hispanic Black females [11.0% (160)]		P-values compared to White females	Non-Hispanic Chinese American females [6.7% (97)]		P-values compared to White females
Age (years)	65.77	(9.07)	64.15	(9.24)	0.06	64.99	(9.99)	0.39	64.39	(9.49)	0.20
Family history of diabetes, % (N)	32.6	(102)	46.2	(80)	0.003	41.3	(64)	0.06	23.2	(22)	0.08
Married/living with a partner, % (N)	59.2	(186)	48.8	(83)	0.03	34.4	(55)	<0.001	70.1	(68)	0.05
Education, % (N)					<0.001			0.59			<0.001
High school or less	26.4	(81)	67.2	(117)		25.0	(40)		47.4	(48)	
Associates	32.2	(102)	21.3	(37)		36.9	(59)		25.8	(25)	
Bachelor's or higher	41.4	(134)	11.5	(20)		38.1	(61)		26.8	(26)	
Annual household income, % (N)					<0.001			<0.001			<0.001
<$25,000	17.1	(51)	49.1	(85)		27.9	(41)		52.1	(50)	
$25,000–$49,999	26.8	(80)	34.7	(60)		37.4	(55)		30.2	(29)	
≥$50,000	56.2	(168)	16.2	(28)		34.7	(51)		17.7	(17)	
Stress, % (N)					0.08			0.04			<0.001
Low	25.1	(79)	34.5	(60)		34.4	(55)		56.7	(55)	
Medium	40.6	(128)	37.4	(65)		30.0	(48)		22.7	(22)	
High	34.3	(108)	28.2	(49)		35.6	(57)		20.6	(20)	
Hypertension medication use, % (N)	32.8	(103)	29.3	(51)	0.43	50.0	(80)	<0.001	24.7	(24)	0.13
Lipid-lowering medication use, % (N)	17.2	(54)	16.7	(29)	0.88	12.7	(20)	0.20	15.5	(15)	0.69
Cigarettes smoking, % (N)					0.01			0.67			<0.001
Never	44.3	(139)	58.6	(102)		47.5	(76)		99.0	(96)	
Former	43.0	(135)	34.5	(60)		38.8	(62)		1.0	(1)	
Current	12.7	(40)	6.9	(12)		13.8	(22)		0.0	(0)	
Alternative healthy eating index (AHEI)-2010	56.31	(10.17)	52.38	(8.82)	<0.001	54.00	(9.70)	0.02	56.46	(7.93)	0.88
Sedentary behavior (MET- hours/day)	4.48	(2.96)	3.52	(2.31)	<0.001	4.91	(2.85)	0.12	3.45	(2.36)	0.001
Exercise (MET-hour/day)	3.37	(3.39)	2.56	(3.03)	0.01	3.34	(3.42)	0.94	3.18	(3.48)	0.64
Body mass index (BMI, kg/m^2)	26.65	(5.23)	29.08	(5.29)	<0.001	29.84	(6.05)	<0.001	23.88	(3.42)	<0.001
Total fat mass (kg)	29.31	(9.65)	31.54	(8.59)	0.01	33.94	(10.98)	<0.001	23.21	(5.49)	<0.001
Abdominal muscle area (cm2)	82.58	(13.62)	81.50	(15.45)	0.43	92.81	(17.42)	<0.001	75.13	(13.10)	<0.001
Abdominal muscle density (HU)	40.29	(4.45)	40.30	(4.96)	0.98	42.78	(5.56)	<0.001	41.63	(4.80)	0.01
Systolic blood pressure (SBP, mmHg)	123.70	(22.01)	128.90	(24.91)	0.02	132.70	(22.37)	<0.001	21.77	(2.21)	0.76

Diastolic blood pressure (DBP, mmHg)	67.22	(9.60)	69.86	(9.66)	0.00	73.36	(10.26)	<0.001	68.88	(8.64)	0.13
Total cholesterol (mg/dl)	203.40	(34.48)	206.50	(35.10)	0.34	195.60	(31.33)	0.02	195.90	(28.96)	0.03
High-density lipoprotein (HDL) cholesterol (mg/dl)	59.96	(16.57)	53.60	(13.81)	<0.001	59.81	(16.28)	0.93	54.96	(12.99)	0.002
Low-density lipoprotein (LDL) cholesterol (mg/dl)	115.40	(28.90)	125.80	(32.81)	<0.001	117.40	(28.58)	0.49	115.40	(28.60)	0.98
Triglycerides (mg/dl)	137.90	(80.92)	138.50	(70.68)	0.94	89.29	(38.85)	<0.001	129.20	(68.57)	0.34

	**Non-Hispanic White males [22.4% (326)]**		**Hispanic males [11.1% (161)]**			**Non-Hispanic Black males [8.4% (122)]**			**Non-Hispanic Chinese American males [7.0% (102)]**		
					**P-values compared to White males**			**P-values compared to White males**			**P-values compared to White males**
Age (years)	64.92	(9.99)	61.60	(9.62)	0.001	64.37	(10.07)	0.60	64.98	(10.57)	0.96
Family history of diabetes, % (N)	22.2	(72)	39.5	(62)	<0.001	45.3	(53)	<0.001	19.0	(19)	0.49
Married/living with a partner, % (N)	76.6	(76)	73.6	(117)	0.47	67.2	(82)	0.04	91.2	(93)	0.001
Education, % (N)					<0.001			<0.001			0.01
High school or less	12.9	(42)	53.4	(86)		23.8	(29)		24.8	(25)	
Associates	26.4	(86)	28.6	(46)		37.7	(46)		19.8	(20)	
Bachelor's or higher	60.7	(198)	18.0	(29)		38.5	(47)		55.5	(56)	
Annual household income, % (N)					<0.001			<0.001			<0.001
<$25,000	8.8	(28)	30.2	(48)		21.8	(24)		40.6	(41)	
$25,000–$49,999	25.9	(82)	39.6	(63)		32.7	(36)		21.8	(22)	
≥$50,000	65.3	(207)	30.2	(48)		45.5	(50)		37.6	(38)	
Stress, % (N)					0.22			0.77			0.03
Low	34.7	(113)	41.0	(66)		31.2	(38)		49.0	(50)	
Medium	37.4	(122)	29.8	(48)		38.5	(47)		31.4	(32)	
High	27.9	(91)	29.2	(47)		30.3	(37)		19.6	(20)	
Hypertension medication use, % (N)	29.5	(96)	28.0	(45)	0.73	37.7	(46)	0.09	22.6	(23)	0.17
Lipid-lowering medication use, % (N)	18.2	(59)	11.2	(18)	0.05	7.4	(9)	0.01	12.8	(13)	0.20
Cigarettes smoking, % (N)					0.55			0.35			0.11
Never	36.7	(118)	35.0	(56)		33.1	(40)		48.0	(49)	
Former	51.9	(167)	50.0	(80)		50.4	(61)		44.1	(45)	
Current	11.5	(37)	15.0	(24)		16.5	(20)		7.8	(8)	
Alternative healthy eating index (AHEI)-2010	52.63	(9.46)	49.17	(9.05)	<0.001	50.86	(10.13)	0.09	54.45	(7.77)	0.05
Sedentary behavior (MET- hours/day)	3.65	(2.35)	3.46	(2.31)	0.41	4.36	(2.78)	0.01	3.40	(2.26)	0.34

Exercise (MET-hour/day)	4.26	(4.50)	3.66	(3.95)	0.15	4.97	(6.87)	0.29	3.17	(3.19)	0.01
Body mass index (BMI, kg/m^2)	27.44	(3.66)	28.43	(4.04)	0.01	27.62	(3.71)	0.65	23.64	(2.77)	<0.001
Total fat mass (kg)	24.51	(6.14)	24.78	(6.61)	0.66	24.19	(6.61)	0.63	16.86	(4.57)	<0.001
Abdominal muscle area (cm2)	123.70	(23.96)	125.70	(22.10)	0.37	136.40	(26.09)	<0.001	111.80	(18.53)	<0.001
Abdominal muscle density (HU)	43.95	(5.32)	44.73	(4.29)	0.08	46.42	(4.57)	<0.001	45.99	(3.65)	<0.001
Systolic blood pressure (SBP, mmHg)	124.70	(18.34)	122.80	(19.48)	0.29	129.90	(20.20)	0.01	123.30	(20.53)	0.52
Diastolic blood pressure (DBP, mmHg)	75.24	(8.77)	75.13	(9.59)	0.90	76.56	(10.17)	0.21	74.12	(9.14)	0.26
Total cholesterol (mg/dl)	189.30	(33.27)	196.10	(34.98)	0.04	187.20	(36.43)	0.55	190.90	(30.71)	0.68
High-density lipoprotein (HDL) cholesterol (mg/dl)	45.90	(11.88)	43.61	(10.18)	0.03	49.45	(13.54)	0.01	47.69	(11.15)	0.18
Low-density lipoprotein (LDL) cholesterol (mg/dl)	118.10	(29.29)	123.10	(31.04)	0.08	119.20	(32.75)	0.72	117.80	(26.91)	0.92
Triglycerides (mg/dl)	128.30	(75.27)	148.60	(77.89)	0.01	92.57	(40.17)	<0.001	126.00	(66.70)	0.78

Data were presented as mean (standard deviation, SD) for continuous variables, and percentage, % (frequency, N) for categorical variables.
When comparing each racial/ethnic minority group to White group, P-values were compared using t-test for continuous variables and $\chi 2$-tests for categorical variables.
When comparing categorical variables, if the frequency < 5, Fisher's exact test was used.

Supplementary Table 12. Decomposition of associations of race and ethnicity with type 2 diabetes (T2D) by visceral fat, removing family history of diabetes from the covariate set

	Natural indirect effect (NIE)					Natural direct effect (NDE)				Total effect (TE)			
	**HR**	**95%CI**		***P***	**Proportion explained (%)**	**HR**	**95%CI**		***P***	**HR**	**95%CI**		***P***
Female													
Hispanic vs. White	**1.23**	**1.13**	**1.40**	**<0.001**	19.4	**2.34**	**2.05**	**2.42**	**<0.001**	**2.94**	**2.57**	**3.05**	**<0.001**
Chinese vs. White	0.95	0.81	1.01	0.10		**2.69**	**2.63**	**3.27**	**<0.001**	**2.44**	**2.38**	**2.96**	**<0.001**
Black vs. White	0.82	0.79	1.03	0.13		**2.19**	**2.05**	**2.62**	**<0.001**	**1.93**	**1.81**	**2.32**	**<0.001**
Male													
Hispanic vs. White	1.05	0.94	1.15	0.50		**1.90**	**1.78**	**2.09**	**<0.001**	**1.95**	**1.83**	**2.14**	**<0.001**
Chinese vs. White	0.81	0.72	1.01	0.06		1.08	0.87	1.18	0.89	**0.91**	**0.74**	**1.00**	**0.04**
Black vs. White	0.95	0.81	1.10	0.54		**1.39**	**1.18**	**1.60**	**0.01**	**1.29**	**1.10**	**1.50**	**0.01**

Natural mediation effects were used to assessed racial and ethnic differences in T2D explained by visceral fat. Bootstrap was used to get 95% confidence interval (CI) and P-values. HR, 95% CI and P-value were reported. Boldface indicated statistical significance (i.e., P<0.05).

Continuous visceral fat (cm^2) was used as the mediator.

Type 2 diabetes (T2D) was regressed on age, sex, race and ethnicity, marital status, education, annual household income, stress, hypertension medication use, lipid-lowering medication use, smoking, alternative healthy eating index (AHEI)-2010, sedentary behavior, exercise, BMI, total fat mass, abdominal muscle area and density, systolic blood pressure (SBP), total cholesterol, high-density lipoprotein (HDL) cholesterol, triglycerides, and visceral fat.

Supplementary Table 13. Characteristics among included and excluded participants

Characteristics		Included participants (n=1457)		Excluded participants (n=490)		P-values
Age (years)		64.58	(9.73)	64.90	(9.41)	0.53
Female, % (N)		51.2	(746)	44.3	(217)	0.01
Family history of diabetes, % (N)		33.1	(474)	52.3	(241)	<0.001
Race and ethnicity						<0.001
	White	44.0	(641)	29.4	(144)	
	Chinese	13.7	(199)	10.8	(53)	
	Black	19.4	(282)	25.3	(124)	
	Hispanic	23.0	(335)	34.5	(169)	
Married/living with a partner, % (N)		64.8	(941)	64.0	(313)	0.94
Education, % (N)						<0.001
	High school or less	32.2	(468)	45.5	(223)	
	Associates	28.9	(420)	27.6	(135)	
	Bachelor's or higehr	39.0	(567)	26.9	(132)	
Annual household income, % (N)						<0.001
	<$25,000	27.5	(389)	38.5	(181)	
	$25,000–$49,999	28.6	(404)	32.8	(154)	
	≥$50,000	44.0	(622)	28.7	(135)	
Stress, % (N)						0.10
	Low	42.1	(613)	36.5	(179)	
	Middle	29.0	(423)	31.6	(155)	
	High	28.9	(421)	31.8	(156)	
Hypertension medication use, % (N)		32.1	(468)	46.4	(227)	<0.001
Lipid-lowering medication use, % (N)		14.9	(217)	19.6	(96)	0.01
Cigerettes smoking, % (N)						0.60
	Never	50.3	(733)	51.0	(250)	

Former	37.2	(542)	35.1	(172)	
Current	12.5	(182)	13.9	(68)	
Alternative healthy eating index (AHEI)-2010	53.40	(9.64)	52.98	(8.60)	0.37
Sedentary behavior (MET- hours/day)	3.96	(2.62)	3.96	(2.53)	0.97
Exercise (MET-hour/day)	3.61	(4.12)	3.10	(3.82)	0.01
Body mass index (BMI, kg/m^2)	27.34	(4.81)	29.59	(5.36)	<0.001
Total fat mass (kg)	26.84	(9.07)	30.14	(10.00)	<0.001
Abdominal muscle area (cm2)	103.60	(28.89)	104.30	(29.24)	0.65
Abdominal muscle density (HU)	42.88	(5.27)	42.39	(5.52)	0.08
Systolic blood pressure (SBP, mmHg)	21.33	(0.56)	22.02	(1.00)	<0.001
Diastolic blood pressure (DBP, mmHg)	72.25	(10.04)	73.32	(10.64)	0.05
Total cholesterol (mg/dl)	196.20	(34.14)	193.20	(33.93)	0.09
High-density lipoprotein (HDL) cholesterol (mg/dl)	52.15	(15.09)	46.92	(13.58)	<0.001
Low-density lipoprotein (LDL) cholesterol (mg/dl)	118.80	(29.99)	116.10	(31.28)	0.10
Triglycerides (mg/dl)	126.50	(72.14)	157.50	(96.08)	<0.001

Data were presented as mean (standard deviation, SD) for continuous variables, and percentage, % (frequency, N) for categorical variables.

P-values were compared using t-test for continuous variables and $\chi2$-tests for categorical variables.

Supplementary Table 14. Decomposition of associations of race and ethnicity with type 2 diabetes (T2D) by visceral fat, applying inverse probability weighting

	Natural indirect effect (NIE)					Natural direct effect (NDE)				Total effect (TE)			
	**HR**	**95%CI**		***P***	**Proportion explained (%)**	**HR**	**95%CI**		***P***	**HR**	**95%CI**		***P***
Female													
Hispanic vs. White	1.23	0.86	1.62	0.25	19.2%	**2.47**	**1.40**	**5.82**	**0.02**	**2.88**	**1.63**	**6.87**	**<0.001**
Chinese vs. White	1.00	0.80	1.26	0.96		**2.95**	**1.06**	**10.36**	**0.03**	2.95	**1.13**	**10.39**	**0.04**
Black vs. White	1.04	0.79	1.31	0.99		**3.55**	**1.69**	**7.70**	**<0.001**	**3.55**	**1.63**	**7.63**	**<0.001**
Male													
Hispanic vs. White	0.97	0.86	1.37	0.48		1.90	0.97	3.74	0.10	2.02	**1.03**	**3.88**	**0.03**
Chinese vs. White	0.82	0.48	1.21	0.31		1.27	0.35	4.00	0.60	1.08	0.30	3.22	0.95
Black vs. White	1.06	0.61	1.18	0.47		1.63	0.86	4.40	0.09	1.50	0.85	3.93	0.16

Natural mediation effects were used to assessed racial and ethnic differences in T2D explained by visceral fat. Bootstrap was used to get 95% confidence interval (CI) and P-values. HR, 95% CI and P-value were reported. Boldface indicated statistical significance (i.e., P<0.05).

Continuous visceral fat (cm^2) was used as the mediator.

Inverse probability weighting was applied to address potential selection bias due to excluding participants.

Type 2 diabetes (T2D) was regressed on age, sex, family history of diabetes, race and ethnicity, marital status, education, annual household income, stress, hypertension medication use, lipid-lowering medication use, smoking, alternative healthy eating index (AHEI)-2010, sedentary behavior, exercise, body mass index (BMI), total fat mass, abdominal muscle area and density, systolic blood pressure (SBP), total cholesterol, high-density lipoprotein (HDL) cholesterol, triglycerides, and visceral fat.
